# The Overlaying of Infants

**Published:** 1904-12-31

**Authors:** 


### The Overlaying of Infants.

The occurrence of an inquest on the body of a young child found dead in bed with one or both of its parents is so frequent an event that it fails to attract public attention. Now and again the total number of such deaths forms a subject for comment and produces a temporary sensation. But for the most part the question is neglected, and the proposals intended to prevent this cause of infant mortality fail to bear any practical result. Possibly the absence of active opposition may in part account for this. Reformers who find a unanimous welcome awaiting their suggestions are apt to be wanting in energy and perseverance, and the stimulus of criticism not only improves them in this respect, but helps their cause by keeping it in the public mind. A recent correspondence in the *Times* gives promise of some assistance of this order, and whatever may be thought of the weight and value of the several contributions the controversy will, we hope, engage general attention. The facts are, on the surface at least, comparatively simple. Young children in large numbers are, without question, found dead in bed, in circumstances which suggest suffocation. In all parts of England and Wales a considerable proportion of such deaths are found by coroners' juries, after medical and other evidence has been heard, to be due to the children having been overlain. Verdicts in accordance with this view are found in London alone in between 500 and 600 cases every year, and the annual average total for the whole country is about 1,800. Thus a great body of medical men, of coroners, and of juries, all having access to the facts of numerous individual cases, accept the view that several hundreds of children every year lose their lives by suffocation in the manner already mentioned.

It is therefore not surprising that proposals have been advanced in favour of an endeavour to meet the position by legislation, and to bring any adult found in bed with a suffocated child within the reach of the criminal law. Dr. Wynn Westcott has argued forcibly in support of this suggestion, and has received support from other coroners. But for some reason or other, and in spite of the fact that according to the general view the lives of some ten thousand infants are sacrificed annually, the proposed legislation has made no advance. It is here that some hope may be founded on the *Times* correspondence, as this, having the flavour of controversy, may attract more general attention. The opposition to what have for the most part been accepted as facts altogether beyond dispute has been headed by Mr. John Troutbeck, the Coroner for Westminster. According to Mr. Troutbeck, though it is true that many children are found dead in bed with their parents, the great majority of these "are not suffocated by any external means, but die perfectly natural deaths." We are not concerned with the implied possibility of suffocation apart from "external means," nor with the proposition that it is "natural" for many hundreds of infants to die when in bed with their parents. Apart from the peculiarities of phrasing, the issue raised is a clear and definite one. On the one side, as we have already indicated, the great majority of experienced authorities say these children are suffocated. Mr. Troutbeck says they are not suffocated. Of course his personal opinion is not advanced as in itself carrying weight on a clinical and pathological subject, and he is careful in his letter to deprecate evidence which is not based on "knowledge and experience in pathology." But he has been able during the last three years, so he tells the readers of the *Times*, to obtain much better evidence than was formerly available, and it is on this that he reposes his confident conclusion. Hence Mr. Troutbeck's appearance in the controversy has merely the interest which is attached to the professions of a convert. He himself will doubtless allow that the important matter is not his opinion, but the evidence of the scientific experts on which it is based. Of the number and weight of these authorities he says nothing, and until information on these points is forthcoming Mr. Troutbeck's contributions to the controversy can have but little value. The curious thing is that the person or persons who are able to provide this "much better evidence" should apparently be content with the *ex cathedrâ* announcements of their disciple in the lay press, and should be so reluctant to bring their evidence where it can be adequately tested. It is certainly to be hoped that those who entertain strong convictions both on the nature of the evil and its remedy will be stimulated to defend their position, and that in this way the truth may be determined and public opinion be educated. If these results follow the appearance of Mr. Troutbeck's personal creed in the columns of the *Times*, all interested in the subject will owe him their thanks.

